# Temporal learning in the suprasecond range: insights from cognitive style

**DOI:** 10.1007/s00426-022-01667-x

**Published:** 2022-03-28

**Authors:** Alice Teghil, Fabrizia D’Antonio, Antonella Di Vita, Cecilia Guariglia, Maddalena Boccia

**Affiliations:** 1 Department of Psychology, “Sapienza” University of Rome, Via dei Marsi, 78, 00185 Rome, Italy; 2 Cognitive and Motor Rehabilitation and Neuroimaging Unit, IRCCS Fondazione Santa Lucia, Rome, Italy; 3 Department of Human Neuroscience, “Sapienza” University of Rome, Rome, Italy

## Abstract

The acquisition of information on the timing of events or actions (temporal learning) occurs in both the subsecond and suprasecond range. However, although relevant differences between participants have been reported in temporal learning, the role of dimensions of individual variability in affecting performance in such tasks is still unclear. Here we investigated this issue, assessing the effect of field-dependent/independent cognitive style on temporal learning in the suprasecond range. Since different mechanisms mediate timing when a temporal representation is self-generated, and when it depends on an external referent, temporal learning was assessed in two conditions. Participants observed a stimulus across six repetitions and reproduced it. Unbeknownst to them, in an internally-based learning (IBL) condition, the stimulus duration was fixed within a trial, although the number of events defining it varied; in an externally-cued learning (ECL) condition, the stimulus was defined by the same number of events within each trial, although its duration varied. The effect of the reproduction modality was also assessed (motor vs. perceptual). Error scores were higher in IBL compared to ECL; the reverse was true for variability. Field-independent individuals performed better than field-dependent ones only in IBL, as further confirmed by correlation analyses. Findings provide evidence that differences in dimensions of variability in high-level cognitive functioning, such as field dependence/independence, significantly affect temporal learning in the suprasecond range, and that this effect depends on the type of temporal representation fostered by the specific task demands.

## Introduction

The acquisition of information concerning the timing of events or responses, following repeated experience, is usually referred to as temporal learning (Church, 2012). This kind of learning is pervasive in everyday life, since many common activities such as preparing to cross the street because we anticipate that the traffic light is going to turn green, or singing our favorite song - crucially depend on the acquired knowledge of the timing of events.

Many studies have investigated temporal learning under different experimental conditions, especially focusing on the learning of subsecond intervals (see Bueti & Buonomano, 2014, for a review). In this range, a robust temporal perceptual learning has been reported, usually indicated by the lowering of temporal discrimination thresholds after multiple trials and training sessions (Bratzke et al., 2012, 2014; Bueti et al., 2012; Karmarkar & Buonomano, 2003; Lapid et al., 2009; Nagarajan et al., 1998; Westheimer, 1999; Wright et al., 1997, 2010; Zhang et al., 2018). Perceptual temporal learning in the subsecond range has also been observed for the discrimination of the speed and isochrony of tone sequences (Ning et al., 2019; van Wassenhove & Nagarajan, 2007), and using interval bisection procedures (e.g. Grondin et al., 2009; Matthews & Grondin, 2012).

Other studies, investigating motor temporal learning, trained participants to reproduce single or multiple subsecond intervals, or more complex spatio-temporal patterns. These studies generally showed an improvement in performance and a reduction of variability (Bartolo & Merchant, 2009; Ivry & Hazeltine, 1995; Laje et al., 2011). Even though many of these motor learning studies involved an explicit training, a gradual improvement in the reproduction of temporal sequences also occurs within single task sessions, and without an explicit instruction to encode the embedded temporal regularity (Brandon et al., 2012; Karabanov & Ullén, 2008; Salidis, 2001; Schultz et al., 2013; Terry et al., 2016; Tillmann et al., 2011; Ullén & Bengtsson, 2003).

Although less investigated, temporal learning seems to occur also in the suprasecond range, i.e. when the duration or timing of events span across seconds. Different studies have provided evidence that the discrimination (Matthews & Grondin, 2012) and reproduction (Warm et al., 1975) of suprasecond durations improve through extensive training. An improvement in suprasecond timing across multiple days of training has been also reported in implicit timing tasks, such as predicting when a drifting bar should reach a specific point after becoming invisible (Sohn & Lee, 2013). Similarly to the subsecond range, suprasecond temporal learning seems to occur also without specific instructions to encode a temporal regularity, as suggested by temporal expectation studies. In discrimination paradigms in which the interval between cues and targets is varied according to different time schedules, indeed, participants usually learn to anticipate the timing of the target, varying the timing of their responses according to the underlying probability distribution (e.g. Bueti & Macaluso, 2010; Herbst & Obleser, 2017).

Notably, relevant differences between participants have been reported in temporal learning studies, with some individuals showing no evidence of improvement also after extensive training (Bartolo & Merchant, 2009; Bueti et al., 2012; Buonomano et al., 2009; Karmarkar & Buonomano, 2003; Laje et al., 2011; Ullén & Bengtsson, 2003; Wright et al., 1997). Nonetheless, individual factors affecting learning of temporal information in these studies, and their relation with specific task demands, have not been investigated to date.

Individual variations in subsecond timing appear to be mainly related to previous training in sensorimotor timing tasks (such as musical training, see for example Cicchini et al., 2012; Matthews et al., 2016; Repp, 2010). Conversely, performance differences in suprasecond timing have been more strongly related to individual differences in personality traits and cognitive functioning (Ogden et al., 2018; Teghil et al., 2019a; Wittmann & Paulus, 2008; Wittmann et al., 2011). Among different relevant dimensions, previous studies highlighted a possible influence of field-dependent/independent cognitive style (FDI) (Witkin, 1977) on temporal processing. FDI is generally conceived as a stable dimension of individual functioning, transversal to different cognitive domains (Witkin, 1977). More specifically, it has been originally defined as an information processing style, that is not affected by experience, and characterizes the way in which a person analyzes the world (Witkin, 1977). Field-independent (FI) individuals usually rely upon an internal frame of reference, and thus are more able to avoid the influence of deceptive information from the external sensory context. Field-dependent (FD) individuals, instead, more likely rely on an external frame of reference, and are more easily mislead by environmental deceptive cues (Witkin, 1977). Among other findings, more FI individuals perform better than FD ones in disambiguating ambiguous sentences (Lefever & Ehri, 1976), and recall less “critical lures” than FD ones in a Deese–Roediger–McDermott paradigm (Corson et al., 2009). Previous studies have also suggested that more FI individuals perform better than more FD ones in suprasecond retrospective timing tasks (Silverman et al., 1961; Teghil et al., 2019a). This advantage has been ascribed to the higher cognitive restructuring skills of FI individuals (Teghil et al., 2019a), that allow them to disengage from external cues, and generally to perform better in tasks in which the material lacks an organization or is ambiguous (Witkin, 1977).

In the present study, we aimed to assess the influence of individual variations in FDI on temporal learning in the suprasecond range. Notably, recent theoretical accounts of time processing suggest that different brain circuits and strategies are involved in timing depending on task and context features (Merchant et al., 2013; Paton & Buonomano, 2018; Wiener et al., 2011). One of these features concerns whether the task involves the self-generation of temporal representations, or whether the timing of the response is determined by the structure of the external environment. Indeed, it has been proposed that different neurocognitive mechanisms mediate time processing when events and responses are timed independently from external cues or variations in perceptual features of the stimuli (internally-based timing), and when time estimation and/or response timing is based on an exogenous sensory signal (externally-cued timing) (Teghil et al., 2019b). This proposal has been supported by meta-analytic evidence, behavioral and neuroimaging studies, showing that the processing of event and response timing in internally-based versus externally-cued conditions entails significant differences in performance (Teghil, Boccia, et al., 2020) and is associated to not-overlapping brain correlates (Teghil et al., 2019b, 2020b, 2020c).

Thus, here we hypothesized that differences in the predisposition towards FDI may affect temporal learning depending on the type of representation needed to perform the task. In more detail, since FI individuals usually perform better in tasks requiring the use of an internal frame of reference, we hypothesized that an advantage for such individuals in temporal learning may be specifically apparent when the task requires to establish and use an internal temporal referent (internally-based timing). Conversely, this advantage should not be apparent when the timing of the response depends entirely on an external referent (externally-cued timing).

To these aims, we developed a novel task assessing temporal learning in two conditions, and testing the acquisition of a timed response based on the repeated experience of a duration (internally-based timing) or of an exogenous sensory pattern (externally-cued timing). In each trial, participants observed a simple stimulus across six repetitions, and were then asked to reproduce the stimulus. Unbeknownst to them, in one condition (internally-based learning, IBL), the total presentation time (i.e. the duration) of the stimulus was the same in each of the six repetitions of a given trial. However, within the total presentation time of the stimulus, a different number of single visual events occurred in different repetitions within the same trial. Thus, in this condition, correct performance in the test phase entailed the extraction and learning of information about the total duration of the stimulation across different repetitions in a trial, whereas the number of single visual events occurring across different repetitions was not relevant. Conversely, in the externally-cued learning (ECL) condition, the stimulus was composed by an equal number of visual events within each trial, although its total duration varied within the trial. Reproducing the stimulus in this condition thus required participants to rely entirely on external visual information. In other words, whereas the IBL condition entailed the formation of a fixed internal temporal referent despite variations in other features of the stimulus, in the ECL condition, the timing of the response had to depend entirely on learning to develop an external referent. Thus, both conditions required participants to perform a response at the right time (see the “Methods” section below). However, in the IBL condition accurate response timing entailed learning the presence of a regularity in the duration of the stimulus (independently from its visual features) and performing a motor response based on an internalized representation of the duration. Instead, in the ECL condition accurate timing involved learning the presence of a regularity in the stimulus visual features (independently from its duration) and performing a motor response when an external signal (i.e. a given number of visual events) was provided.

Since the two conditions required participants to perform a timed response, both of them involved temporal processing. Nevertheless, the two conditions differed in terms of the type of temporal representation required to achieve a correct performance. Whereas the IBL condition required to develop a representation of the duration of events independently from external sensory features, the ECL condition required to perform a timed motor response based on a specific sensory signal, similarly to what happens for internally generated and externally triggered movements (Teghil et al., 2019b).

Finally, it has been shown that the modality in which the knowledge of the timing of events is demonstrated—i.e. through a motor response or through a perceptual discrimination judgment—affects performance (Bueti & Walsh, 2010; Bueti et al., 2008). Thus, as a secondary aim, we tested the possibility that expressing temporal learning through a motor response vs. a perceptual judgment interacts with the type of temporal representation (internally-based vs. externally-cued) and with the predisposition towards FDI. More specifically, since only the IBL condition should require the self-generation of a temporal representation, it is possible that performance in this condition may be improved when this knowledge is expressed though an entirely motor response. Conversely, when the task requires to time the response based on an external cue (ECL), an advantage may be present for perceptual reproduction.

In line with previous studies reporting evidence of temporal learning in the suprasecond range (Bueti & Macaluso, 2010; Sohn & Lee, 2013; Warm et al., 1975), we focused on a range between approximately 3 and 15 s. Different studies have indeed provided evidence that mechanisms mediating timing in this range are different from those involved in subsecond timing (Gooch et al., 2011; Hayashi et al., 2014; Macar et al., 2002; Wiener et al., 2010; Wittmann et al., 2007), and the processing of temporal information in this range is strongly associated to high-level cognitive processing (Badouin et al., 2006; Lewis & Miall, 2003). Thus, based on findings that FDI affects performance in cognitively controlled tasks such as environmental navigation (Boccia, Piccardi, et al., 2017) and visual search (Guisande et al., 2007), we expected temporal learning in this range to be affected by individual differences in FDI.

To summarize, we hypothesized that FI individuals should perform better than FD ones specifically when the task entails the self-generation of a temporal representation (IBL). Also, as a secondary aim, we tested whether individual variations in FDI interacts with the modality (i.e. motor vs. perceptual reproduction) in which the task is performed.

## Methods

### Participants

A total number of 39 participants (19 females; mean age: 23.051 years, SD 1.621; mean education = 16.487 years, SD 1.412) took part in the study. This sample size was in line with previous studies investigating individual differences in complex cognitive processes (Jia et al., 2014; Sulpizio et al., 2017; Teghil et al., 2019a). Furthermore, the sample size for the comparison between internally-based (IBL) and externally-cued (ECL) learning was estimated a priori using G*Power (Version 3.1.9.2; Faul et al., 2007). The effect size for the power analysis was derived from an independent sample of 16 participants (8 females; mean age: 23.875 years, SD 2.061; mean education: 16.25 years, SD 1.39), who took part in a pilot study, as follows. In the pilot study, participants were asked to perform the same task used in the main study, in two possible conditions, namely IBL and ECL (10 trials for condition, arranged in two blocks) (see below). Average absolute error scores (calculated using the formula |(Reproduced duration – Target duration)/Target duration|)^1^ in IBL and ECL, as well as their correlation and standard deviation, were used to determine the effect size in G*Power. The resulting Cohen *d*_z_ was 0.77, and the estimated sample size to achieve a statistical power higher than 95% when contrasting IBL and ECL, considering an alpha level of 0.01 (two-tailed), was 34. Thirty-nine participants were enrolled considering possible technical issues and dropouts.

All participants were right-handed, and had normal or corrected-to-normal vision. None of them had a previous or current history of neurological and/or psychiatric disorders, as assessed by means of an informal interview prior to the experimental testing session. All participants scored within the normal range in the Raven’s Coloured Progressive Matrices (Basso et al., 1987). The study was designed in accordance with the principles of the Declaration of Helsinki and was approved by the ethical committee of Fondazione Santa Lucia, Rome. Informed consent was obtained from all individual participants included in the study.

### Assessment of FDI

In line with previous literature (Boccia, Vecchione, et al., 2017, 2019; Teghil et al., 2019a), we assessed individuals’ predisposition towards FDI using the paper-and-pencil version of the Embedded Figures Test (EFT) (Witkin et al., 1971). This test shows an overall high reliability, with reported coefficients in different samples of young adults ranging between 0.76 and 0.89 (Witkin et al., 1971). Moreover, performance on the paper-and-pencil version of the EFT correlates with that on the Rod and Frame Test (Gardner et al., 1960; Pizzamiglio & Carli, 1973), supporting the convergent validity of the EFT in assessing individual predisposition towards FDI.

In brief, the EFT requires participants to locate simple geometric shapes that are embedded within more complex configurations. Test material is composed by two sets of 12.9 × 7.7 cm cards, on which simple shapes and complex figures are printed. In each trial, the experimenter presents one complex figure for 15 s. Then, the card showing the complex figure is covered, and a simple shape is shown for 10 s. After that, the experimenter presents the complex figure again, and the participant is required to locate the simple shape embedded into the complex figure as quickly as possible. For each trial (total *N* = 12), score corresponds to the total number of seconds required to locate the simple shape. If the simple shape is not located within 3 min, the item is scored as wrong, and the participant is automatically given a score of 180 s. The total EFT score corresponds to the average solution time across the 12 trials. Thus, lower scores indicate stronger predisposition towards field-independence.

### Temporal learning task

**Fig. 1:**
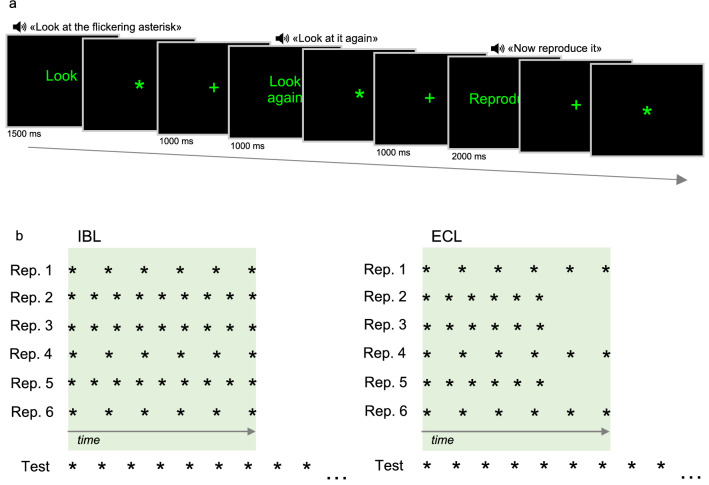
**a** A trial of the temporal learning task. At the beginning of each trial, an instruction appeared on screen for 1500 ms, instructing participants to observe the stimulus; the same instruction was also presented auditorily. In each trial, the stimulus (a flickering asterisk on a black background) was presented six times. After the first stimulus presentation, the instruction “Look” (“Osserva” in Italian) was replaced by the instruction “Look again” (“Osserva di nuovo” in Italian), presented both visually and auditorily, for the remaining five presentations (only one of the five presentations is showed in the figure). After the six presentations of the stimulus, the instruction “Reproduce” (“Riproduci”), presented both visually and auditorily, marked the beginning of the test phase, and a fixation cross appeared on screen. In the perceptual reproduction condition, the fixation cross was replaced by the flickering asterisk after 500 ms. Participants were instructed to press the spacebar key to interrupt the presentation of the asterisk when they thought they had reproduced it. In the motor reproduction condition, participants were asked to press the spacebar key to start the presentation of the asterisk, and to hold the key until they thought they have reproduced it. **b** Schematic illustration of the variation in stimulus parameters in the IBL and ECL conditions. In each trial of the temporal learning task, the six repetitions of the stimulus could either involve the switching between two different numbers of flickers (IBL condition) or between two different durations (ECL condition). Thus, in the IBL condition, in each trial the six presentations of the stimulus were characterized by a fixed duration, but two different numbers of flickers (leftward panel). Instead, in a single trial of the ECL condition, the total presentation time of the stimulus could take two different duration values, but the number of flickers was fixed (rightward panel)

We developed a computerized task to assess internally-based (IBL) and externally-cued (ECL) temporal learning. The experiment was developed as a within-subject design, with all participants performing all conditions (see below). A trial of the experimental paradigm is shown in Fig. 1a. In each trial, participants were asked to observe a green asterisk (RGB 0/1/0), flickering on a black background. In the learning phase of each trial, the flickering asterisk was presented six times. Then, in the test phase, participants were instructed to reproduce the stimulus they observed. Before each of the six repetitions of the stimulus in the learning phase, a recorded female voice instructed participants to observe the stimulus. The same voice announced the starting of the test phase of each trial. The duration and flickering parameters of the stimulus in the learning phase were varied in order to create two conditions, as follows. In trials of the IBL condition, each of the six repetitions of the stimulus had a fixed duration; the flickering frequency of the stimulus, instead, took two different values within each trial (one value in three repetitions, and a different one in the other three). Thus, since the total presentation time of the stimulus in each repetition of the learning phase of a trial was fixed, but its flickering frequency varied, the total number of flickers of the asterisk was different between one half of the repetitions and the other (Fig. 1b, leftward panel). In the ECL condition, the flickering frequency of the stimulus also took two different values in each trial, but the duration of the repetitions was arranged such as that the stimulus always flickered a fixed number of times in a given trial. In other words, although in a given trial the stimulus was presented for two different durations (three repetitions for each duration), the number of times it flickered was fixed (Fig. 1b, rightward panel). In each trial of both conditions, the two parameters (number of flickers/duration) in the six repetitions followed a fixed randomized order. In both conditions, the flickering frequency of the stimulus in the test phase was different from that in the six presentations of the learning phase (Fig. 1b). Thus, participants were forced to base the timing of their response entirely on acquired information about the duration (IBL) and number of flickers (ECL) of the stimulus to correctly perform the task. Notably, no reference to temporal reproduction or to time was made when instructing participants to perform the task, since they were simply asked to “reproduce the stimulus they observed”. This ensured that they were not biased to focus on a specific dimension of the stimulus during the two conditions.

Each participant performed two 5-trial blocks for each condition (IBL and ECL), one requiring reproduction in the motor (*M*) and one in the perceptual modality (*P*). In the *M* condition, participants were required to press the spacebar key on a laptop to start the reproduction phase and to display the stimulus, and to hold the key until they judged they have reproduced the stimulus. In the *P* reproduction condition, the stimulus was presented automatically, and participants were asked to press the spacebar key to interrupt its presentation when they judged they had reproduced it (Bueti & Walsh, 2010; Bueti et al., 2008). Thus, the combination of the factors “Condition” and “Reproduction modality” configured 4 conditions: IBL, motor (IM) and perceptual (IP), and ECL, motor (EM) and perceptual (EP).

Duration, frequency and number of flickers parameters in each Condition and trial are reported in Table 1. The two Conditions (IBL and ECL) were matched for mean target duration (IBL: M 8430 ms, SD 2188.886; ECL: *M* 9549 ms, SD 3075.417; *t*_18_ = 0.937, *p* = 0.361), mean number of flickers (IBL: *M* 11.150, SD 1.684; ECL: *M* 10.600, SD 1.897; *t*_18_ = − 0.686, *p* = 0.502), and mean hertz during the presentation (IBL: *M* 2.781 Hz, SD 0.696; ECL: *M* 2.768 Hz, SD 1.102; *U* 41, *p* 0.495)^2^ and test phases (IBL: *M* 2.336 Hz, SD 0.706; ECL: *M* 2.471 Hz, SD 1.011; *U* = 46, *p* = 0.760) (see footnote 2). Within each Condition, the two blocks were also matched for the same parameters (all *p*s > 0.05, two-tailed; see Table 2 for full details about means, SDs and test statistics of the comparisons between blocks).

**Table 1:** Stimulus parameters in each trial of the temporal learning task

Condition	Block	Trial N°	Flickers N° (1)	Flickers N° (2)	Total duration (ms) (1)	Total duration (ms) (2)	Hz (1)	Hz (2)	Hz (test)	Target duration (ms)
IBL	1	1	12	6	8400		2.857	1.429	1.905	8400
	1	2	18	6	9000		4.000	1.333	2.000	9000
	1	3	18	9	10,800		3.333	1.667	2.222	10,800
	1	4	14	7	5600		5.000	2.500	3.333	5600
	1	5	18	9	7200		5.000	2.500	3.333	7200
	2	1	18	6	10,800		3.333	1.111	1.667	10,800
	2	2	12	6	4800		5.000	2.500	3.333	4800
	2	3	15	5	7500		4.000	1.333	2.000	7500
	2	4	16	8	11,200		2.857	1.429	1.905	11,200
	2	5	15	5	9000		3.333	1.111	1.667	9000
ECL	1	1	11		8690	15,950	2.532	1.379	1.786	11,700
	1	2	10		7900	14,500	2.532	1.379	1.786	10,800
	1	3	8		2800	4560	5.714	3.509	4.348	11,200
	1	4	12		9480	12,120	2.532	1.980	2.222	7200
	1	5	8		2800	11,600	5.714	1.379	2.222	9000
	2	1	13		4550	7410	5.714	3.509	4.348	13,440
	2	2	12		12,120	14,760	1.980	1.626	1.786	12,320
	2	3	9		9090	11,070	1.980	1.626	1.770	10,170
	2	4	10		5700	12,300	3.509	1.626	2.222	5980
	2	5	13		7410	15,990	3.509	1.626	2.222	3680

Trial N°: order of the trial in the block (please note that the two blocks of each condition are named 1 and 2 only for descriptive purposes, since their presentation order was balanced across participants); flickers N° (1) and (2): the two alternating numbers of flickers of the stimulus across the six repetitions in the learning phase; total duration (1) and (2): the two alternating total presentation durations of the stimulus across the six repetitions in the learning phase; Hz (1) and (2): the two alternating flickering frequencies of the stimulus across the six repetitions in the learning phase; Hz (test): flickering frequency of the stimulus in the test phase; target duration: target duration in the specific trial

**Table 2:** Means (standard deviations) of stimulus parameters in the two blocks of the temporal learning task in the IBL and ECL conditions. Test statistics for the comparisons between parameters in the two blocks are also reported. IBL internally-based learning, ECL externally-cued learning. ^a^Non-parametric analyses were performed because the assumption of the normality of the distribution was violated

	Block 1	Block 2	Test statistics
IBL			
Target duration (ms)	8200 (1949.359)	8660 (2616.868)	*t*_8_ = − 0.315, *p* = 0.761
Number of flickers	11.7 (1.956)	10.6 (1.342)	*t*_8_ = 1.037, *p* = 0.33
Hertz (presentation phase)	2.962 (0.744)	2.601 (0.675)	*U* = 9, *p* = 0.455^a^
Hertz (test phase)	2.5587 (0.7164)	2.1143 (0.6971)	*U* = 9, *p* = 0.165^a^
ECL			
Target duration (ms)	9040 (3557.977)	10,058 (2823.831)	*t*_8_ = − 0.501, *p* = 0.630
Number of flickers	9.8 (1.788)	11.4 (1.816)	*t*7.998 = − 1.403, *p* = 0.198
Hertz (presentation phase)	2.865 (1.177)	2.671 (1.15)	*U* = 10.5, *p* = 0.672^a^
Hertz (test phase)	2.473 (1.071)	2.469 (1.073)	*U* = 11.5, *p* = 0.827^a^

The pairing between block, condition and reproduction modality, as well as the presentation order of the four blocks, was balanced across participants according to a Latin square design. Immediately before each block, participants performed two training trials, with the same structure of the task, and requiring reproduction in the same modality as the following block (in training trials there was no recorded voice, and instructions were provided directly by the experimenter, who also paced each presentation of the stimulus in the learning phase and the starting of the reproduction phase).

Reproduced duration (i.e. the time elapsed between the starting of the stimulus presentation in the test phase and that of the spacebar press in the *P* condition, and the time elapsed between the starting of the keypress in the test phase and that of the key release in the *M* condition) was recorded. All the stimuli were generated and presented on a laptop, using the Cogent Toolbox (Cogent, http://www.vislab.ucl.ac.uk/Cogent/) for MATLAB (Mathworks).

### Procedure

Participants were tested individually, in a well-lit and quiet room. All tasks were performed in a single experimental session, lasting ~ 1 h. The administration order of the EFT and of the temporal learning task was counterbalanced across participants.

### Analyses and results

Statistical analyses were performed using SPSS (IBM SPSS Statistics 20) and R 3.6.2 (R Core Team, 2019). Participants were divided into an FI and an FD group according to a median split of EFT scores (averaged solution times). Participants with scores lower than the median (31.12) were classified as FI, while participants with scores higher than the median were classified as FD. The proportion of females and males did not differ significantly in the FI and FD groups (*χ*^2^ = 3.092, *p* = 0.079). The average EFT score was not significantly different between male and female participants (*U* 126, *p* = 0.074, two-tailed) (see Footnote 2) FD and FI participants did not differ significantly for age (*U* 175.5, *p* = 0.669, two-tailed)^2^ or education (*U* 154.5, *p* = 0.301, two-tailed)^2^﻿.

### Temporal learning task: effect of target duration

Trials in which the reproduced duration was longer than 3 × (target duration) or shorter that (target duration)/3 were excluded from further analyses. These represented 1.154% of the total number of trials.

To investigate whether participants were able to perform the task in both conditions (IBL and ECL), we first performed two one-way ANOVAs to compare reproduced durations within each condition, independently from the reproduction modality. Since preliminary analyses showed that reproduced durations were not normally distributed, we performed one-way robust ANOVAs on 20% trimmed means with 5000 bootstrap samples, using the WRS2 package for R (Mair & Wilcox, 2020).

**Fig. 2:**
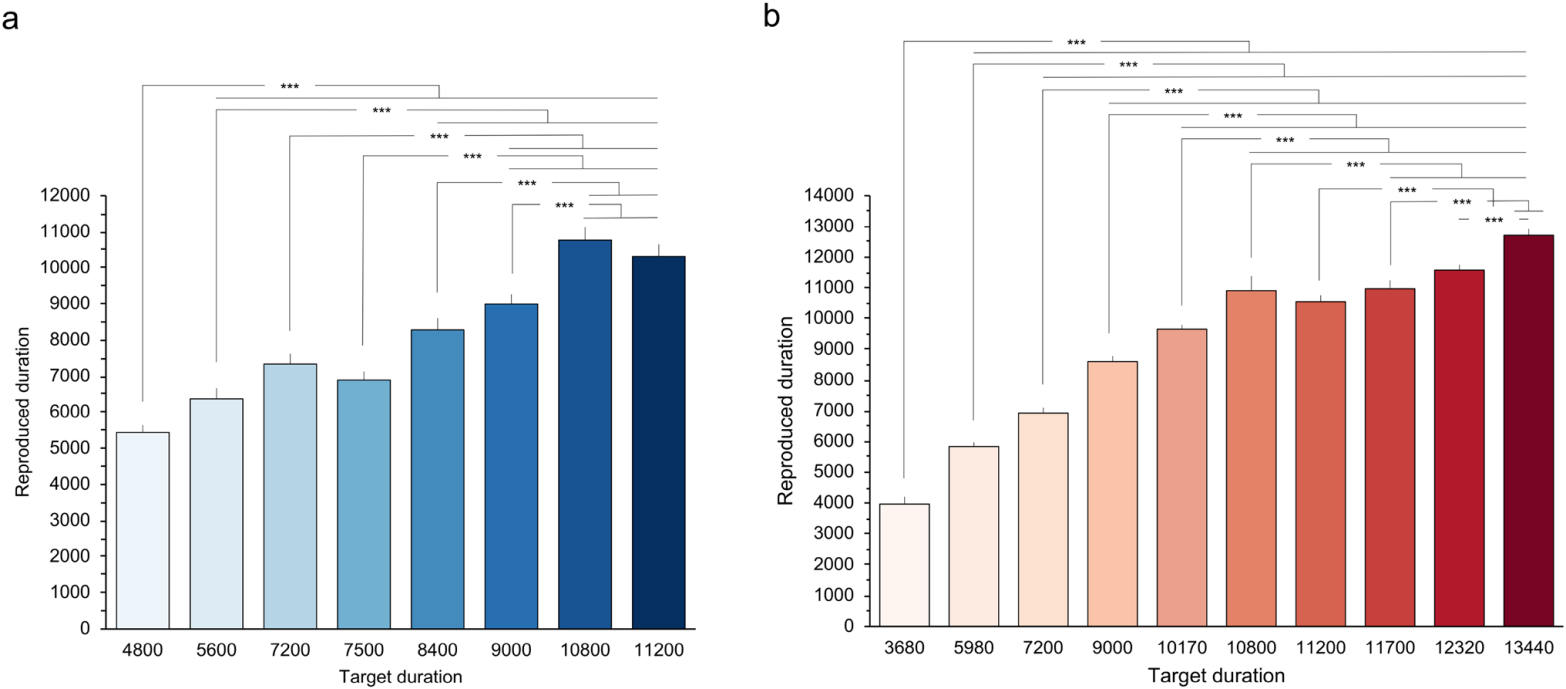
Mean reproduced duration (ms) for each target duration in **a** the IBL, and **b** the ECL condition. Significant differences are marked with asterisks. ***p < 0.001

In the IBL condition, the effect of Target duration was significant (*F*_t_ = 58.623, *p* < 0.001, *ξ* = 0.692). Post-hoc comparisons with Bonferroni correction showed that reproduced times for the 4800 ms target duration were significantly different from those reproduced for the 7200, 7500, 8400, 9000, 10,800 and 11,200 ms target durations (*p* < 0.001), but not from those reproduced for the 5600 ms target duration (*p* = 0.029). Reproduced times for the 5600 ms target duration were also not significantly different from those for the 7200 (*p* = 0.005) and 7500 ms (*p* = 0.02) target durations, but were significantly different from those reproduced for the 8400, 9000, 10,800 and 11,200 ms target durations (*p* < 0.001). Reproduced times did not differ between the 7200 ms and the 7500 ms (*p* = 0.946) and 8400 ms (*p* = 0.006) target durations, but for 7200 ms were significantly different than those reproduced for the 9000, 10,800 and 11,200 ms target durations (*p* < 0.001). Reproduced times for the 7500 ms target duration were significantly different from those for the 9000, 10,800 and 11,200 ms target durations (*p* < 0.001), but not from those for the 8400 ms target duration (*p* = 0.005). There was also a significant difference between reproduced times for the 8400 ms and the 10,800 ms and 11,200 ms target durations (*p* < 0.001), but not between reproduced times for 8400 and 9000 ms (*p* = 0.143). Finally, there was a significant difference between reproduced times for 9000 and 10,800 and 11,200 ms (*p* < 0.001), but not between those for 10,800 and 11,200 ms (*p* = 0.484) (Fig. 2a).

The effect of Target duration was also significant in the ECL condition (*F*_t_ = 3077.91, *p* < 0.001, *ξ* = 0.869). Post-hoc comparisons with Bonferroni correction showed that all comparisons between target durations were significant (*p* < 0.001), except those between the 10,800 and 11,200 (*p* = 0.062), the 11,700 and 11,700 (*p* = 0.001), and the 11,700 and 12,320 ms target durations (*p* = 0.003) (Fig. 2b).

### Effect of FDI and reproduction modality

For each trial in each condition and reproduction modality, we calculated an absolute error score (es) for each participant, using the following formula:

$${\text{es}} = \left| {\frac{{{\text{Reproduced duration}} - {\text{Target duration}}}}{{\text{Target duration}}}} \right|$$

Preliminary analyses showed that absolute error scores in the four conditions did not meet the assumptions of parametric analyses, since the residuals of the DVs were not normally distributed. Also, Levene’s test indicated unequal variances for IP condition in the FD and FI groups (*F*_1,37_ = 6.402, *p* = 0.016). Thus, non-parametric or robust statistical methods were used in the following analyses.

**Fig. 3:**
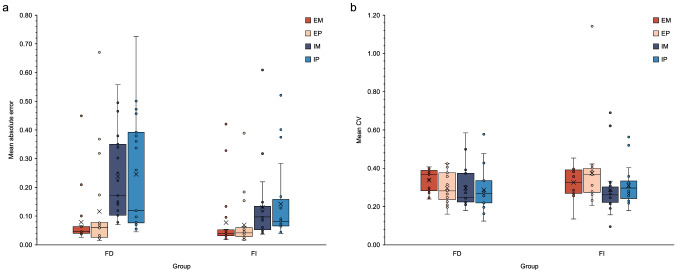
**a** Results of the analyses comparing absolute error scores in the four conditions of the temporal learning task between groups. **b** Results of the analyses comparing the coefficient of variation (CV) in the four conditions of the temporal learning task between groups. *EM* externally-cued learning, motor reproduction, *EP* externally-cued learning, perceptual reproduction, *IM* internally-based learning, motor reproduction, *IP* internally-based learning, perceptual reproduction, *FD* field-dependent group, *FI* field-independent group

To compare absolute error scores in the four conditions of the task between FD and FI participants, we performed a Welch–James test with Approximate Degrees of Freedom (Welch ADF) using the welchADF package for R (Villacorta, 2017), which allows to deal with non-normally distributed data and heterogeneous distributions in mixed-factorial designs. Bootstrapping was used to calculate p values for main effects and interactions. The Welch–James test was performed with condition (IBL, ECL) and modality (M, P) as within-subjects factors, and Group (FI, FD) as between-subjects factors. We found a significant effect of Condition (WJ_1,13.92_ = 23.229, *p* = 0.006), with higher error scores in IBL (*M* 0.187, SD 0.153) than in ECL (*M* 0.085, SD 0.111). Also, the effect of Group was significant, with higher error scores in the FD (*M *0.170, SD 0.217) compared to the FI group (*M* 0.101, SD 0.167) (WJ_1,13.57_ = 6.876, *p* = 0.033). There was no significant effect of Modality (WJ_1,16.50_ = 0.035, *p* = 0.838). We found a significant effect of the Group × Condition interaction (WJ_1,13.92_ = 6.178, *p* = 0.026, bootstrap critical value for Family-Wise Error Rate control = 5.081, effect size standardized via square root of the average of cell variances = − 2.472, 95% CI [− 4.167, − 0.142]), with FI participants performing significantly better than FD ones in the IBL (FI: *M* 0.136, SD 0.124; FD: *M* 0.242, SD 0.165; *U*  109.5, *p* = 0.024, two-tailed, r = -0.362) but not in the ECL (FI: *M* 0.073, SD 0.094; FD: *M* 0.097, SD 0.127; *U* 133.5, *p* = 0.112, two-tailed, *r* = − 0.254) condition (Mann–Whitney’s tests were used to specify the origin of the interaction effect, see Jiménez-Urbieta et al., 2020; Aguirre et al., 2019, for similar procedures). All the other interaction effects were not significant (Modality × Condition: WJ_1,17.01_ = 0.155, *p* = 0.686; Group x Modality: WJ_1,16.50_ = 0.119, *p* = 0.743; Group x Modality x Condition; WJ_1,17.01_ = 0.008, *p* = 0.931) (Fig. 3a).

Additional analyses were performed to compare the variability in the four conditions of the task between FD and FI participants. For each participant in each interval, we calculated the coefficient of variation (CV) of reproduced times, dividing the standard deviation by the mean reproduced time. Descriptive statistics showed that the residuals of the DVs were not normally distributed. Thus, a Welch–James test was performed with Condition (IBL, ECL) and Modality (M, P) as within-subjects factors, and Group (FI, FD) as between-subjects factors. The effect of Condition was significant, with the CV significantly higher in the ECL (*M* 0.334) than in the IBL condition (*M* 0.296) (WJ_1,17.27_ = 14.86, *p* < 0.001, bootstrap critical value for Family-Wise error rate control = 4.204, effect size standardized via square root of the average of cell variances = 2.273, 95% CI [0.506, 3.930]). There was no other significant effect (modality: WJ_1,20.35_ = 0.00693, *p* = 0.952; Group: WJ_1,19.83_ = 0.0001908, *p* = 0.990; modality × condition: WJ_1,22.45_ = 2.535, *p* = 0.107; group × modality: WJ_1,20.35_ = 1.03, *p* = 0.304; group × condition: WJ_1,17.27_ = 0.8287, *p* = 0.392; group × modality × condition; WJ_1,22.45_ = 3.09, *p* = 0.561) (Fig. 3b).

**Fig. 4:**
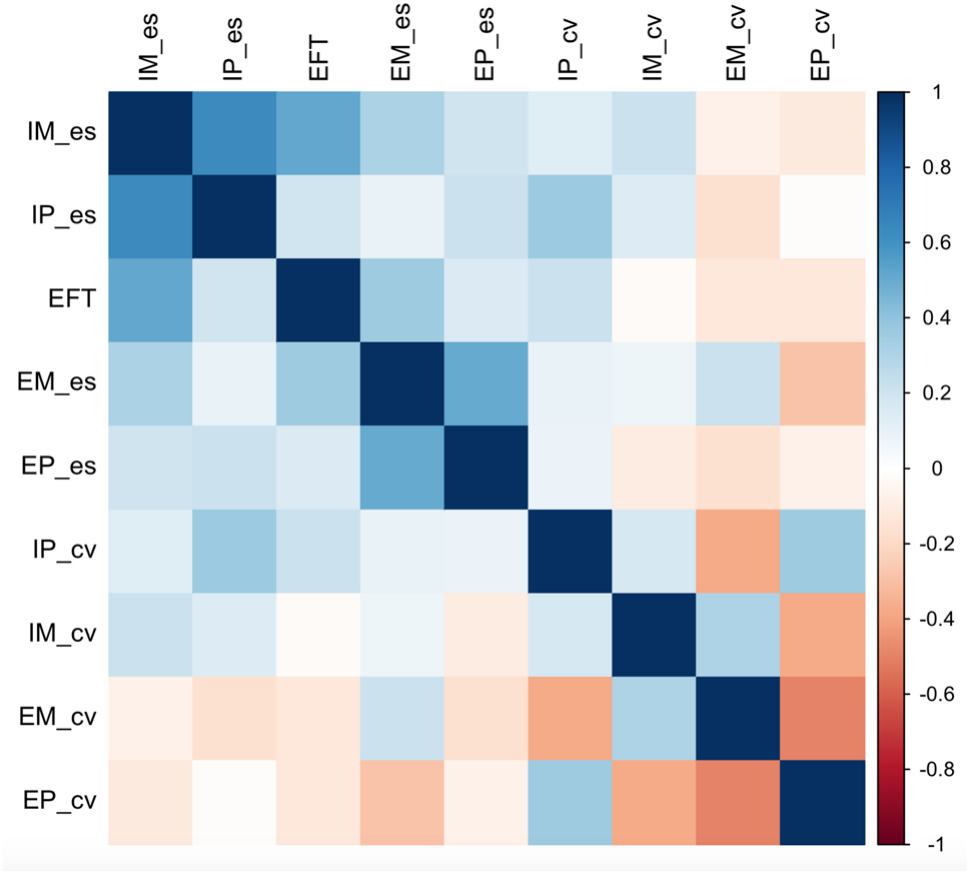
Correlation plot showing the association between EFT scores and absolute error scores and coefficients of variation in the externally-cued learning, motor reproduction, externally-cued learning, perceptual reproduction, internally-based learning, motor reproduction, and internally-based learning, perceptual reproduction conditions. *EFT* EFT scores, *es* absolute error score, *cv* coefficient of variation, *EM* externally-cued learning, motor reproduction, *EP* externally-cued learning, perceptual reproduction, *IM* internally-based learning, motor reproduction, *IP* and internally-based learning, perceptual reproduction

To further assess the relation between the degree of FDI and performance in the temporal learning task, we performed two-tailed Spearman’s correlations between EFT scores and absolute error scores and CVs in the four conditions of the task. A sensitivity analysis performed using G*Power (Version 3.1.9.2) (Faul et al., 2007), using a two-tailed test of significance, an alpha level of 0.05 and a statistical power higher than 80% in a bivariate correlation analysis, showed a sensitivity to detect an effect size of 0.418. Results of the correlations analyses are reported in Table 3 and shown in Fig. 4. EFT scores were significantly correlated with absolute error scores in the IM and in the EM condition, whereas the correlation with scores in the IP and EP conditions were not significant. We also found a significant correlation between absolute error scores in the IM and IP conditions as well as those in the EM and EP conditions. The CV in the EM condition significantly correlated with that in the EP and IP conditions; the CV in the EP condition was significantly correlated with that in the EM and IP conditions. Finally, a significant correlation was found between error scores and CV in the IP condition. When a Bonferroni correction for multiple comparisons was applied, setting alpha at 0.05/45 = 0.0011, the only significant correlations were between absolute error scores in the two IBL and ECL conditions, and, notably, between EFT scores and absolute error scores in the IM condition.

**Table 3:** Spearman’s correlation coefficients between EFT scores and absolute error scores and coefficients of variation in the four conditions of the task

	EFT	EM_es	IM_es	EP_es	IP_es	EM_cv	EP_cv	IM_cv	IP_cv
EFT	1	0.352*	0.519**	0.153	0.199	− 0.126	− 0.124	− 0.02	0.216
EM_es	0.352*	1	0.316	0.509**	0.095	0.212	− 0.289	0.078	0.096
IM_es	0.519**	0.316	1	0.208	0.638***	− 0.079	− 0.115	0.216	0.137
EP_es	0.153	0.509**	0.208	1	0.214	− 0.163	− 0.077	− 0.1	0.081
IP_es	0.199	0.095	0.638***	0.214	1	− 0.167	− 0.017	0.14	0.363*
EM_cv	− 0.126	0.212	− 0.079	− 0.163	− 0.167	1	− 0.492**	0.309	− 0.374*
EP_cv	− 0.124	− 0.289	− 0.115	− 0.077	− 0.017	− 0.492**	1	− 0.374*	0.351*
IM_cv	− 0.023	0.078	0.216	− 0.104	0.14	0.309	− 0.374*	1	0.172
IP_cv	0.216	0.096	0.137	0.081	0.363*	− 0.374*	0.351*	0.172	1

*EFT* EFT scores, *es* absolute error score, *cv* coefficient of variation, *EM* externally-cued learning, motor reproduction, *EP* externally-cued learning, perceptual reproduction, *IM* internally-based learning, motor reproduction, *IP* and internally-based learning, perceptual reproduction

*p < 0.05; **p < 0.01; ***p < 0.001

### Summary of main findings

Overall, we found a main effect of Condition (IBL, ECL), with higher error scores in the IBL, and higher variability (coefficient of variation) in the ECL condition. Moreover, in the IBL condition FI participants performed better than FD ones, showing lower error scores. This latter result was further corroborated by Spearman’s correlations, even when a stricter correction for multiple comparisons was applied.

## Discussion

Here we assessed the effects of individual differences in FDI on temporal learning, in two conditions, namely when learning the timing of a response entails building up an internal referent of a target duration (internally-based timing), and when the timing of the response is driven by learning an exogenous sensory pattern (externally-cued timing). Moreover, we investigated the possible interaction between these factors, and the type of response required by the task (motor vs. perceptually driven timed response). To these aims, we developed a novel temporal learning task, requiring participants to learn and reproduce a stimulus based on a representation of its duration independent from external referents (IBL), or based on an external regular signal (ECL). The task was performed requiring either a sustained motor response, or a perceptual judgment (Bueti & Walsh, 2010; Bueti et al., 2008).

Results of the analyses on mean reproduced durations (i.e. the timing of the response) in IBL and ECL showed that participants could overall perform the task in both conditions. Thus, they were able to learn to base the timing of their response both on a regularity in the stimulus duration, and on a regularity in the stimulus visual features. These findings are in line with evidence that healthy individuals are generally able to extract relevant organizational patterns from an apparently ambiguous stream of stimulation, in both the auditory and visual domain, and in a variety of contexts and settings (see Aslin & Newport, 2012, for a review). In the domain of timing, specifically, this effect has been previously demonstrated for temporal order statistics (Baker et al., 2014; Schapiro et al., 2012), as well as for motor learning of subsecond temporal patterns (Brandon et al., 2012; Karabanov & Ullén, 2008; Salidis, 2001; Schultz et al., 2013; Terry et al., 2016; Tillmann et al., 2011). Present results extend previous findings, suggesting that a rapid and effective learning of temporal regularities following repeated exposure also occurs in the second range, in line with indirect evidence provided by temporal expectation studies (Bueti & Macaluso, 2010; Herbst & Obleser, 2017, among others).

Analyses comparing mean error scores in the four conditions of the task between FD and FI participants showed that the deviation from the target duration was overall higher in the IBL condition. These findings are thus in line with previous evidence that providing an external cue for timing improves performance compared to when this cue is not available (Teghil et al., 2020b; Teghil, Boccia, et al., 2020). Analyses, however, also showed a significant interaction between the type of temporal representation needed to solve the task (internally-based vs. externally-cued) and the predisposition towards FDI. Whereas accuracy was comparable between FD and FI individual when response timing relied on an acquired external sensory representation, this was not true for IBL, in which FI participants were significantly more accurate than FD ones (Fig. 3a). This latter result is thus consistent with the hypothesis that a stronger predisposition towards FDI provides a specific advantage in temporal learning when task demands entail the establishment of an internal temporal referent.

Whereas ECL required to perform a timed response based on a representation of a regularity in a sensory feature not related to events duration, IBL required to perform a similar response based on a representation of a regularity in the duration of experienced events. Since performance in IBL selectively required to develop an internal temporal referent, differences between FD and FI participants in this condition may be possibly understood in the light of classical information-processing models of time perception. These models generally link psychological time to the activity of an “internal clock”, composed by a pacemaker, an accumulator, a memory stage—including a working memory and a reference memory component—and a comparator mechanism (e.g. Allman et al., 2014; Zakay & Block, 1997). As mentioned in the Introduction, an influence of FDI has been reported in tasks involving cognitive restructuring, such as visuospatial and navigational learning (Boccia, Vecchione, et al., 2017; Tascón et al., 2017), but also semantic and episodic memory tasks (Corson et al., 2009; Spiro & Tirre, 1980). Present results thus suggest that differences between FD and FI individuals in cognitive restructuring may have affected the formation of a long-term temporal referent during IBL. Notably, memory for durations has been shown to strongly rely on hippocampal-related processing (see Clewett et al., 2019, for a review): lesions to the fimbria-fornix impair reference memory in rats (Meck et al., 2013) and hippocampal activity is sensitive to the duration of individual events within sequences (Barnett et al., 2014; Thavabalasingam et al., 2019). Thus, an intriguing possibility is that FI individuals may have engaged more effectively in hippocampal-mediated processes—such as pattern separation—during the encoding phase of the IBL condition, leading to an improved representation of target durations in specific trials. Although such a possibility was not tested in the present study, it would be in line with evidence that FI individuals engage more strongly than FD ones in item-specific processing during episodic memory encoding (Corson et al., 2009).

Previous evidence of a better performance of FI compared to FD individuals in timing tasks has been mainly reported using retrospective estimation paradigms (Silverman et al., 1961﻿; Teghil et al., 2019a). Thus, these studies did not allow to completely rule out the possibility that such an advantage was specific to the type of paradigm employed (e.g. retrospective verbal estimation). Present results suggest that differences between FD and FI in timing tasks may be also observed in prospective paradigms. More in detail, finding that FI individuals specifically performed better in the internally-based condition of the temporal learning task supports the possibility that the advantage of a stronger predisposition towards field-independence is specifically apparent in timing tasks in which the use of an internal frame of reference is needed (Teghil et al., 2019a; Witkin, 1977). Interestingly, if this were the case, such differences should be apparent also in prospective timing tasks involving durations longer than those tested in the present study, since the use of an internal frame of reference could help more FI individuals to disambiguate overlapping memory traces of experienced durations. Overall, further research is thus warranted to understand the relation between individual variability in cognitive restructuring and long-term memory, and how they interact in temporal learning.

Findings of an interaction between task condition (IBL vs. ECL) and variations in FDI also shed some light on how individual differences in FDI affect cognitive functioning. In this vein, it has been proposed that differences in cognitive functioning between FD and FI individuals may be mainly conceived as differences in inhibition and resistance to interference (Goode et al., 2002; Imanaka et al., 2017; Jia et al., 2014). Present results, however, are not entirely consistent with these accounts. Indeed, if the main feature distinguishing FI and FD individuals were the stronger competence of the former in extracting relevant task information despite interference or the presence of distractors, an advantage for FI participants in temporal learning should have been observed both when ignoring an irrelevant external sensory signal (IBL) and when ignoring an irrelevant duration (ECL).

Here we also highlighted a significant correlation between scores on the EFT, and performance in the IM condition, pointing to a possible specific relation between the degree of reliance on an internal frame of reference, and the ability to perform a motor timed response based on an internal representation of elapsed time. This latter finding suggests that the need to demonstrate learning of temporal information through a motor response further enhances the advantage of FI participants in the IBL condition. Interestingly, this result is in line with recent proposals that the explicit representation of the duration of events is built based on motor behavior (Coull & Droit-Volet, 2018; Coull et al., 2016), suggesting that timing improvements due to motor processing (Gavazzi et al., 2013; Wiener et al., 2019; see also De Koch et al., 2021, for a review) could be particularly apparent when the task requires to build up an inner representation of time independently from external cues. In this vein, recent studies providing evidence of a tight coupling between timing and motor control also suggest that using a motor response to reproduce a time interval without external cues may improve precision, possibly by allowing the simulation of the target interval (Wiener et al., 2019). Further studies, however, would be needed to better understand the relation between motor processing and different types of temporal representation.

Analyses on the CV further showed that performance was more variable in the ECL than in the IBL condition, independently from the reproduction modality. This latter finding is consistent with the possibility that timing in internally-based and externally-cued conditions depends on different mechanisms (Teghil Boccia, & Guariglia, 2019b; Teghil et al., 2020b, 2020c; Teghil, Boccia, et al., 2020), and more generally with recent accounts suggesting that different strategies may be used to time events in a flexible and task-dependent manner (Paton & Buonomano, 2018; Wiener & Kanai, 2016). Also, this result provides further information on the relation between FDI and temporal learning, showing that differences in the extent to which individuals tend to rely on an internal frame of reference may be apparent in the accuracy rather than precision of temporal learning. It is worth noting, however, that the use of counting strategies was not explicitly controlled for in the present study. Whereas there is evidence that suprasecond timing accuracy may be similar when using counting strategies or not, the use of such a chronometric strategy to keep track of elapsing time heavily affects timing performance when assessed through the CV (Hinton & Rao, 2004; Thönes & Hecht, 2017). Thus, the finding of a higher variability in ECL compared to IBL could be due to a reduction of the variability associated to the latter condition, driven by the use of a counting strategy. Here we choose not to control for counting strategies in the IBL condition to leave participants free to choose their preferred strategy to perform the task in both conditions. However, future studies should compare variability in temporal learning across internally-based and externally-cued conditions, also controlling for counting strategies.

It is also important to point out that, in the present study, target durations in the IBL and ECL conditions were not matched on a single trial basis. Since the order of presentation of blocks was balanced across participants (see “Methods” section), setting the same target durations in the two conditions would have introduced a possible bias, since participants experiencing the IBL condition first could have been led to use knowledge about previous target durations also during ECL. Thus, our design was not aimed and did not allow to directly compare learning of a specifically timed response between the two conditions. Since IBL and ECL were matched for mean target duration and flickering parameters, however, effects related to individual differences in FDI are unlikely to be due to differences in the required timing of the target motor responses in the two conditions.

Finally, here we did not highlight any effect of the reproduction modality. This suggests that, in both the IBL and the ECL conditions, participants developed an abstract temporal representation, that was used in both response modalities. This finding is apparently in contrast with previous evidence that the purpose for which temporal information is encoded (i.e. motor reproduction vs. perceptual discrimination) significantly affects timing performance (Bueti & Walsh, 2010; Bueti et al., 2008). However, the abovementioned studies focused on the subsecond or peri-second range. It is widely acknowledged that subsecond and suprasecond timing rely on different brain mechanisms: whereas the former has been associated to intrinsic and modality-specific neural processing (Goel & Buonomano, 2014; Karmarkar & Buonomano, 2007), timing in the suprasecond range is more dependent on cognitive processing, relying on the activity of a wide cortical network that includes prefrontal and parietal regions (Lewis & Miall, 2003). Thus, although this hypothesis remains speculative and further research will be needed, it is likely that possible effects related to the motor or perceptual nature of the task may be specifically apparent in subsecond timing paradigms.

## Conclusions

Here we showed for the first time that individual differences in field-dependent/independent cognitive style significantly affect the acquisition of temporal knowledge, in line with previous evidence that the degree to which individuals tend to rely on an internal frame of reference affects performance in different cognitive domains, including time processing (Boccia, Piccardi, et al., 2017; Boccia, Vecchione, et al., 2017; Silverman et al., 1961; Teghil et al., 2019a). Also, our findings provide evidence that such individual differences interact with the type of representation required by the task, since they exert a differential effect on temporal learning depending on whether it relies on an internal representation of time, or on an external sensory cue. Overall, present results suggest to further investigate the role of individual differences in learning of temporal information, and the way in which such dimensions of individual variability interact with task demands and with the range of durations tested.

## Data Availability

The script for the temporal learning task and participants’ anonymized data are available from the corresponding author on reasonable request.

## Footnotes

1 Please note that, whereas in the IBL condition the target duration was equal to the fixed presentation time of the stimulus in that specific trial, the same was not true for the ECL condition, in which the target duration in each trial corresponded to the time elapsed between the starting of the test phase, and the time in which participants were expected to interrupt the test phase based on the number of visual stimuli presented (see below). We choose to focus analyses on reproduced durations (and related measures) to allow the comparison between participants’ performance in the two conditions.

2 Non-parametric analyses were performed since the assumption of the normality of the distribution was violated.
